# An old drug, a classic signature

**DOI:** 10.1007/s12471-026-02065-3

**Published:** 2026-07-24

**Authors:** Francisco Rodrigues dos Santos, Mariana Duarte Almeida, Davide Moreira, João Miguel Santos, Inês Fiúza Pires

**Affiliations:** Unidade Local de Saúde Viseu Dão Lafões, Viseu, Portugal

## Answer

**Fig. 1:**
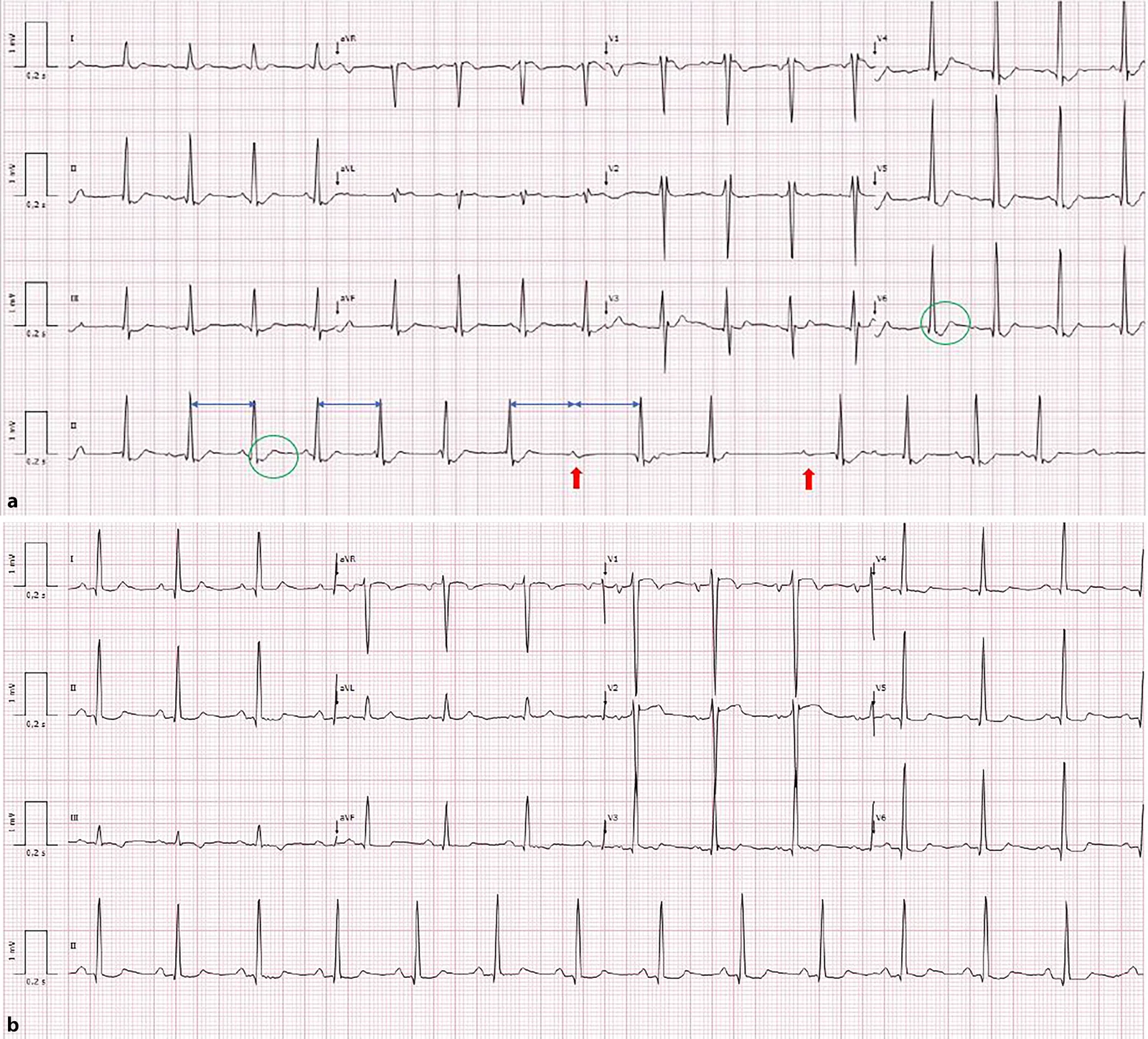
**a** Classic digoxin toxicity findings, red arrow—auricular activity dissociated from QRS; blue arrow—regular RR—junctional rhythm; green circle—shortened QTc with “scooped” morphology. **b** ECG after stabilization and digoxin levels normalization

The electrocardiogram (Fig. 1a) shows an ectopic junctional rhythm with isorhythmic atrioventricular dissociation, a shortened QTc interval, and diffuse ST-segment depression with the characteristic ‘scooped’ or *Salvador Dalí moustache *morphology, findings highly suggestive of digoxin toxicity [1]. Serum levels confirmed to be markedly elevated at 11.5 ng/mL. Although digoxin was not in her recent discharge summary, previous exposure was confirmed, and further investigation revealed a suicide attempt by voluntary ingestion. The clinical presentation, including gastrointestinal symptoms, altered mental status, acute kidney injury, and hyperkalemia, supported the diagnosis of severe digoxin intoxication.

Digoxin use is characterized by a narrow therapeutic window. Toxicity presents with a broad spectrum of clinical and electrocardiographic manifestations. While there is no linear relationship between serum concentration and symptoms, intoxication is most frequently observed at levels > 2.0 ng/mL. Management focused on a three-pillar strategy: normalization of electrolytes (specifically targeting hyperkalaemia), aggressive supportive care and the administration of digoxin-specific antibody fragments. Following treatment, the patient showed rapid biochemical improvement. At discharge, ECG (Fig. 1b) demonstrated sinus rhythm with complete resolution of abnormalities, confirming the causal relationship.

Cardiac involvement is potentially lethal, encompassing virtually any arrhythmia. Typical ECG findings include PR prolongation, QT/QTc shortening, and characteristic scooped ST-segment depression. Enhanced automaticity and atrioventricular nodal suppression may result in junctional rhythms, atrioventricular dissociation, and ventricular ectopy [2]. Of particular concern is the risk of malignant ventricular arrhythmias, notably bidirectional polymorphic ventricular tachycardia. Early recognition of these ECG patterns is critical, as prompt treatment with Digoxin-specific antibody fragments can be life-saving [3].

## Data Availability

All data supporting the findings of this work are included in the article.
